# The Impact of Educational Sessions on Anxiety Levels among Women Undergoing Caesarean Section: A Quasi-Experimental Study

**DOI:** 10.3390/ejihpe14020022

**Published:** 2024-02-06

**Authors:** Fatimah Alsufyani, Nouran Katooa, Ahlam Al-Zahrani, Ohood Felemban, Hanan Badr, Hala Thabet

**Affiliations:** 1 Faculty of Nursing, Taif University, Taif 21944, Saudi Arabia; fatimahammar@tu.edu.sa; 2 Faculty of Nursing, King Abdulaziz University, Jeddah 21589, Saudi Arabia; nkuttouaha@kau.edu.sa (N.K.); ofelemban@kau.edu.sa (O.F.); habadr@kau.edu.sa (H.B.); 3 Faculty of Nursing, Mansoura University, Mansoura 35516, Egypt; hamohammed@kau.edu.sa

**Keywords:** preoperative anxiety, elective caesarean delivery, preoperative education

## Abstract

Although the Caesarean section (CS) is considered a harmless surgery, it has various complications. Women scheduled for elective CSs often have high levels of anxiety due to a lack of knowledge. The aim of this quantitative quasi-experimental study was to determine the relationship between preoperative educational sessions and anxiety levels among women undergoing CSs. The study was conducted at the antenatal unit in the King Faisal Medical Complex (KFMC) in Taif, Saudi Arabia, using a structured interview questionnaire, the State–Trait Anxiety Inventory (STAI), and satisfaction interviews. A total of 50 pregnant women participated in this study, who were divided into two groups: 25 participants in the intervention group and 25 in the control group. Most participants (92%) in the intervention group had low anxiety levels following educational sessions, and 96% of the participants were very satisfied with the preoperative information they had been given. Women in the control group (again, 92%) had high anxiety levels, and there was a significant difference in the anxiety levels of the intervention and control groups (*p* ≤ 0.5) after the educational sessions. Providing proper preoperative education about CSs can reduce preoperative anxiety, improve patient outcomes, and enhance patients’ involvement in their care and decision-making.

## 1. Introduction

Nowadays, Caesarean section (CS) rates are increasing worldwide and are currently 25% in Europe, 32% in North America, and 41% in South America [1]. About 18.5 million CSs are performed annually around the world [2], and in Saudi Arabia, the CS rate has dramatically increased [3,4,5]. CS surgery is linked with various fears related to the birth experience, anxiety, age, labour pain, previous uterine scarring, extended labour, foetal asphyxia, breech presentation, urogenital laceration, and various other maternal complications [1,6,7]. Although CS is considered a harmless surgery, it has various complications, including urinary tract injury, bladder or uterus infection, heavy blood loss, uterine wounds, infection, placenta previa, hysterectomy, placenta accrete, and many others [8,9].

A study reported that 72.7% of women who were scheduled for elective CSs had high levels of preoperative anxiety and fear—higher than those of women scheduled for general surgical procedures [10]. Several factors may contribute to preoperative anxiety, such as previous postoperative pain and nausea, fear of death, adverse experiences with anaesthesia, cultural diversity, the extent and type of surgery, age, gender, past adverse birth experiences, clinical or obstetric complications, harm to the infant, and damage to the mother [10,11].

Anxiety may negatively affect surgery outcomes for women and their babies. In the mother, the effects include tachycardia, arrhythmia, hypertension, increased levels of pain, pain management difficulties, slower wound healing, increased anaesthetic requirements, increased nausea and vomiting, longer hospitalisation, increased costs of care, and death [12]. Also, severe anxiety may result in mothers feeling greatly fatigued [12]. Anxiety may impact the mother’s vital signs, resulting in elevated heart rate and blood pressure [13]. A mother’s anxiety can also have a significant impact on her baby’s mental and physical development, reducing the capacity of her blood to carry oxygen and nutrients to the baby’s developing organs [12].

Preoperative education delivered by healthcare providers can effectively reduce patients’ preoperative anxiety, increase their involvement in their care, decrease possible postoperative complications, and enhance their involvement in decision-making [14]. It plays a major role in enhancing mothers’ awareness and knowledge, reducing their anxieties about surgery and allowing them to remain calm and positive [15]. Preoperative education may reduce analgesic requirements, decrease surgery complications, increase outcome satisfaction, and shorten recovery times [16,17]. Various preoperative educational strategies may be used, involving different teaching methods and multiple media, including written and visual materials such as posters, booklets, videos, photo albums, and medical equipment demonstrations [16]. Moreover, it is important to ensure that mothers receive information about CSs early and frequently to support their understanding of the procedure [18]. It is known that preoperative information and education given to women undergoing CS may lead to better care, recovery, and outcomes. Hence, healthcare providers should implement effective educational interventions to reduce preoperative anxiety levels and improve patient outcomes. The aim of this study was to determine the relationship between preoperative educational sessions and anxiety levels among women undergoing CSs.

## 2. Methods and Materials

### 2.1. Study Design

The study was based on a quantitative quasi-experimental research design. It was conducted at the King Faisal Medical Complex (KFMC) in Taif, Saudi Arabia. The researchers recruited 50 pregnant women undergoing CSs and divided them equally into an intervention group and a control group. The inclusion criteria included pregnant women who were admitted to the antenatal unit, scheduled for elective CS, with no medical and/or surgical complications, aged between 20 and 35 years old, willing to participate in the study at a gestational age of 37 weeks or more, undergoing CS for the first time, and speaking and understanding the Arabic language. The study exclusion criteria included women with psychiatric illnesses and/or who were on any antianxiety or antidepressant medication. The Faculty of Nursing at King Abdulaziz University (KAU) and the Ministry of Health (MOH) approved the research proposal for this study. In addition, permission for data collection was obtained from KFMC. The participants gave their written consent to participate in the study. They were informed about the study’s aim and objectives, that their participation was voluntary, and that they had the right to withdraw at any time during the study without affecting the care provided to them.

### 2.2. Study Tool

A structured questionnaire was used for data collection. It consisted of two parts: Part I aimed to collect data about the participants’ sociodemographic characteristics, obstetric histories, and vital signs; Part II included the State–Trait Anxiety Inventory (STAI-Y1–Y2) and questions regarding the women’s satisfaction.

### 2.3. Vital Signs Assessment

The researchers recorded the participants’ vital signs, including the pulse rate, blood pressure, temperature, and respiratory rate. The vital signs were measured twice for the control group: once before routine care, about 3–4 h before CS, and again after routine care, about 15–30 min before CS. The vital signs were also measured twice for the intervention group: once before the educational sessions, about 3–4 h before CS, and again after the educational sessions, about 15–30 min before CS.

### 2.4. STAI-Y1–Y2

The STAI-Y1–Y2 scale was adapted from Spielberger [19] and modified to suit the aim and objectives of the study. The scale includes two sections: Y1 measures state anxiety (how the participant feels ‘right now’), and Y2 measures trait anxiety (how the participant feels ‘generally’). A high total score indicates intense fear, approaching terror, and panic; a medium total score indicates a moderate level of tension and worry; and a low total score reflects calmness. A score of more than 44 on the STAI was taken to mean significant anxiety, and the patients were categorised as having high anxiety (STAI score > 44) or low anxiety (STAI score ≤ 44).

### 2.5. Women’s Satisfaction

In the intervention group, data were collected via interviews to measure their satisfaction with the educational sessions provided to them, using questions such as ‘Are you satisfied with the preoperative information given in the educational booklet?’.

### 2.6. Validity and Reliability of the Instrument

To evaluate the content validity of the tool, the researchers asked three nursing faculty members who specialised in maternity and women’s health in the faculty of Nursing at KAU, to review the questionnaire. The experts reviewed the tool for its clarity, comprehensiveness, and appropriateness for the research aim. The reliability of the tool was tested using Cronbach’s alpha coefficients. The Cronbach’s alpha for STAI-Y1–Y2 was 0.87, which confirmed its excellent reliability.

### 2.7. Data Collection Procedure

The study included 50 pregnant women who were purposefully selected to participate in the study. The data collection was conducted over eight months, starting at the beginning of December 2019 and continuing until the end of July 2020, with information gathered three days per week in the morning. The data collection process consisted of two phases.

*Phase I: Assessment and Planning*

Pregnant women who met the criteria and agreed to participate in the study were divided into two equal groups: 25 in the intervention group and 25 in the control group. The aim and objectives of the study were explained to each participant, and the participants gave their informed consent to participate prior to the data collection.

*Phase II: Implementation*

Interviews were conducted with all study participants in both the intervention and control groups. The pregnant women in both groups were interviewed before their CS upon admission to the antenatal unit, and their vital signs were measured twice. For the control group, one interview was conducted before routine care, about 3–4 h before CS, and another after routine care, about 15–30 min before CS. For the intervention group, one interview was taken before the educational sessions, about 3–4 h before CS, and again after the educational sessions, about 15–30 min before CS.

The participants’ anxiety levels were measured by a researcher for the intervention group twice (before the educational sessions, 3–4 h before CS, and after the educational sessions, 30–40 min before CS) using the STAI. The anxiety levels of the control group were also measured twice (3–4 h before CS and after routine hospital care, 30–40 min prior to CS) using the STAI. The control and intervention groups received routine hospital care, including nurses asking the mothers to change into hospital gowns and provide urine samples. In addition, an intravenous (IV) line was placed into each mother’s arm or hand to administer necessary fluids and medications.

A researcher conducted individual educational sessions for the intervention group on the days of their surgeries. Each session was used to discuss symptoms with the mother, explain the process and what to expect during the CS and postoperative periods, and help her fully express her fears. The duration of each session ranged from 30 to 60 min.

### 2.8. Educational Sessions

A simple CS educational booklet was designed in the Arabic language, arranged in a logical sequence for expectant mothers, and supplemented with illustrations to facilitate learning. It covered the definition of CS, information about the types of CS and incisions, and details concerning surgical wound care and complications.

The general objective of formulating the educational booklet was to improve the expectant mothers’ knowledge of CS, decrease their levels of anxiety about the process, and correct any misconceptions about CS. The booklet was reviewed by three experts involved in maternity and women’s health nursing at a nursing faculty at KAU in Jeddah, Saudi Arabia. In addition, the booklet was used to guide and support interactions between the researcher and the pregnant women during the educational sessions.

Each pregnant woman was given simple written, illustrated instructions regarding the objectives, outlines, and expected outcomes of the intervention. 

### 2.9. Statistical Design

The data were collected, coded, categorised, and tabulated using tests of statistical significance, such as percentages, *t*-tests, chi-squared tests, McNemar tests, and Fisher’s exact or Monte Carlo correction tests, to identify the relationships between the variables using the Statistical Package for the Social Sciences (SPSS^®^) programme, version 22. Significance was set at *p* ≤ 0.05 for all tests.

## 3. Results

A total of 50 pregnant women participated in this study. They were divided into two groups: 25 participants in the intervention group and 25 participants in the control group.

### 3.1. Demographic Data

The mean ages of the intervention and control groups were 28.56 ± 4.13 and 29.80 ± 4.62 years, respectively. There were no significant differences between the sociodemographic data characteristics of the control and intervention groups. Details are provided in Table 1.

### 3.2. Participants’ Obstetric Data

More than half (52%) of the pregnant women in the intervention group had had 1–2 pregnancies compared to roughly two-thirds (60%) in the control group, while more than a quarter had had 3–4 pregnancies in both groups. There were no statistically significant differences between the groups regarding their gravida (*p* = 0.538), parity (*p* = 0.866), number of living children (*p* = 1.000), previous methods of delivery (*p* = 1.000), number of abortions (*p* = 0.388), or whether they received information about surgery (*p* = 0.258). Other details are mentioned in Table 2.

### 3.3. Participants’ Sources of Anxiety

**Figure 1:**
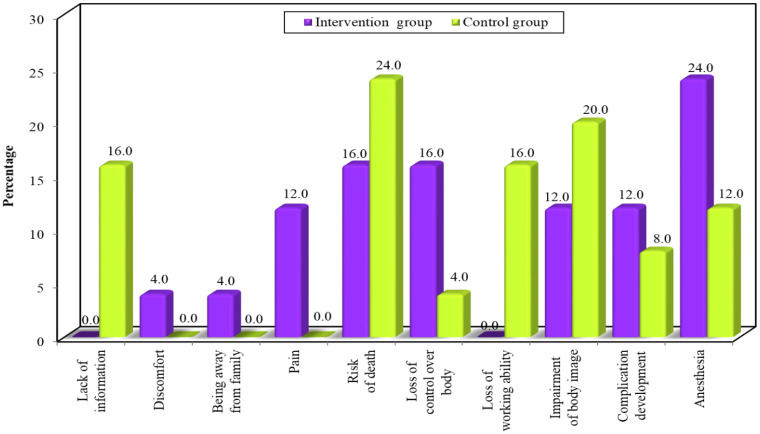
Sources of anxiety among the intervention and control groups.

The participants in the intervention group reported that their sources of anxiety related to the following: fear of anaesthesia (24%), loss of control (16%), risk of death or complication development (16%), impairment of body image (12%), pain (12%), being away from family (4%), and discomfort (4%). The participants in the control group reported that their sources of anxiety were related to the risk of death (24%), impairment of body image (20%), loss of working ability (16%), lack of information (16%), anaesthesia (12%), complication development (8%), and loss of bodily control (4%). Figure 1 illustrates the sources of anxiety.

### 3.4. Medical Reasons for Undergoing CS

**Figure 2:**
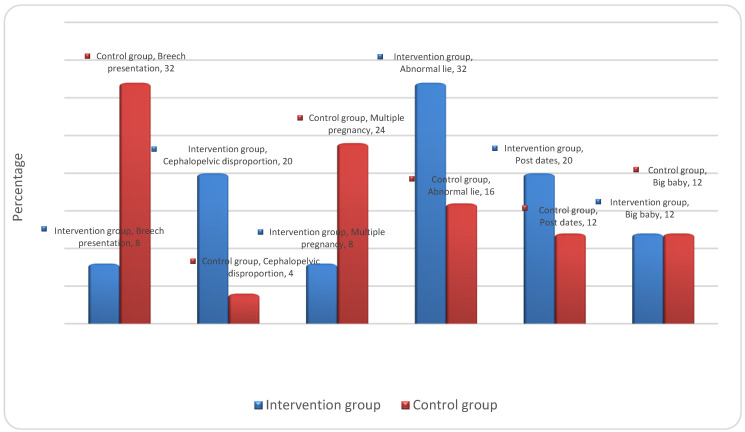
Medical reasons for CSs in the intervention and control groups.

The main reasons for the participants in the intervention group undergoing CSs were abnormal lying (32%), followed by cephalopelvic disproportion (20%), postdates (20%), large babies (12%), multiple pregnancies (8%), and breech presentation (8%). The reasons for the participants in the control group undergoing CSs were breech presentation (24%), multiple pregnancies (32%), abnormal lie (16%), postdates (12%), large babies (12%), and cephalopelvic disproportion (4%). Figure 2 illustrates the medical reasons for CS.

### 3.5. Reasons for Choosing CS

**Figure 3:**
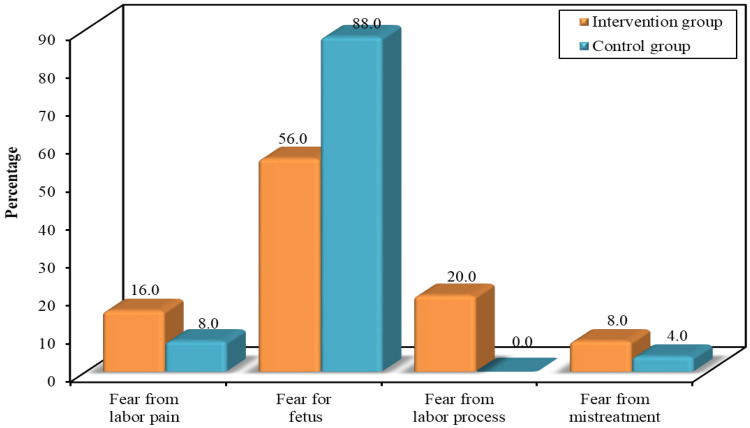
Reasons for choosing elective CSs for the intervention and control groups.

Figure 3 reveals that the main reason for most of the participants (88%) in the control group (88%) choosing elective CSs was fear for the foetus, whereas the rest of the participants reported fear of pain and fear of mistreatment (8% and 4%, respectively). More than half (56%) of the participants in the intervention group reported fear for the foetus, followed by fear of the labour process, fear of labour pain, and fear of mistreatment (20%, 16%, and 8%, respectively).

### 3.6. Anxiety Levels and Vital Signs among the Intervention and Control Groups

There was a statistically significant difference in the overall means for the anxiety levels and vital signs of the participants (*p* ≤ 0.5) in the intervention group after the educational sessions and among those in the control group after routine care. There was an insignificant difference in the anxiety levels and vital signs of the participants in the intervention and control groups before the educational sessions and routine care. However, regarding body temperature, there was a statistically insignificant difference between the intervention and control groups before and after the educational sessions and routine care (Table 3).

### 3.7. STAI Scores among the Intervention and Control Group Participants

The mean STAI scores among the participants in the intervention group before the educational sessions and for the control group before routine care were 65.68 ± 10.69 and 68.0 ± 9.88, respectively, with no statistically significant differences. The mean STAI scores for the intervention group after the educational sessions and for the control group after routine care were 38.28 ± 9.31 and 69.36 ± 10.59, respectively, with significant differences at *p* ≤ 0.5 (Table 4).

### 3.8. STAI Levels among the Intervention and Control Group Participants

Regarding overall anxiety levels, most of the pregnant women (92%) had low anxiety levels in the intervention group after the educational sessions. The same percentage (92%) of the control group had high anxiety levels. There was a significant difference between the groups before routine care and after the educational sessions, at *p* ≤ 0.5 (Table 5).

### 3.9. Participants’ Satisfaction with the Educational Sessions

All the participants (100%) with high overall anxiety levels were satisfied with the educational sessions provided before the CS. About two-thirds (65.2%) of the pregnant women with low overall anxiety were satisfied, and more than a quarter (34.8%) were very satisfied. In addition, all the participants (100%) with high overall anxiety levels were satisfied with the preoperative information provided, compared to more than two-fifths (43.5%) of the participants with low overall anxiety (Table 6).

### 3.10. Correlations between Mean Overall Anxiety Levels and Vital Signs between the Intervention and Control Groups

Table 7 shows that there were significant differences at *p* ≤ 0.5 between the intervention and control groups for mean overall anxiety level, pulse, respiration, and systolic and diastolic blood pressure after the intervention, but there was an insignificant difference between the groups before the intervention. However, there was an insignificant difference in body temperature between the groups before and after the intervention.

### 3.11. Correlations among Mean Overall Anxiety, Post-Educational Session, Sociodemographic Characteristics, and Obstetric Data between the Intervention and Control Groups

Table 8 shows the correlations among the mean overall anxiety, sociodemographic characteristics, and obstetric data for the intervention and control groups. The results revealed that post-intervention, there were significant differences between the two groups regarding age, education level, occupation, gravity, parity, and number of abortions (*p* < 0.001). There was no significant difference between the two groups regarding marital status or income.

## 4. Discussion

The study results showed that the intervention and control groups differed significantly regarding age, education level, occupation, gravity, parity, and number of abortions, but there was no significant difference regarding marital status. A study conducted by [12] also found significant differences in levels of anxiety, depending on the participants’ education levels. Furthermore, aligning with the current study, a study found that women with low education levels (i.e., primary and secondary education) tended to have moderate-to-high levels of anxiety compared to women with high education levels (i.e., university education) [12]. Another study showed that a low level of education was the major factor increasing anxiety during pregnancy compared to women with a high education level [20]. This may be because an educated patient finds it easier to understand a preoperative educational programme and obtain adequate information about the surgery. The purpose of preoperative education is to prepare women for surgery and increase their awareness of what can happen after an operation. It also helps women play an active role in their recovery and gives them a sense of control.

The current study provided preoperative educational sessions about CSs for pregnant women using a guided booklet to relieve their anxiety before their operations. We found that inadequate CS information was a major source of anxiety among pregnant women overall, highlighting the value of preoperative educational sessions. A study reported that women experiencing CSs for the first time displayed significantly higher anxiety levels due to a lack of information and experience [21]. In addition, the risks of death, loss of working ability, and impairment of body image were major sources of anxiety among the participants in the control group. However, in the intervention group, pain, a lack of bodily control, and anaesthesia were the main concerns among the pregnant women. Thus, a lack of information caused anxiety and fear among the participants who did not receive educational sessions before surgery. Similar studies conducted by [7,22] showed that a lack of information and a lack of time for nurses to educate patients caused anxiety among women. A study by [10] showed that the anxiety levels of women undergoing general anaesthesia were higher than those of women undergoing local anaesthesia, a finding which is supported by another study [23]. Also, a previous study reported that pregnant women with high levels of anxiety before surgery may request general anaesthesia because they cannot deal with the challenges of being awake [10]. In contrast, another study emphasised that most pregnant women become anxious due to possible neonatal development disorders and their infants’ responses to their anxiety [12]. Thus, it can be argued that factors related to a lack of information and the participants’ own body issues were the main causes of anxiety among the participants.

The main reason for undergoing CSs among the intervention group participants was an abnormal lie, and in the control group, it was a breech presentation. In Mylonas and Friese’s review study [24], abnormal lie and the presentation of the baby were the main reasons for CS, while a study conducted by [6] showed that the major obstetric indications for CS were obstructed labour, foetal distress, and abnormal presentation. An abnormal foetal position can lead to impossible vaginal delivery, requiring a CS. In the current study, most of the participants in the control group reported that the main reason for choosing elective CSs was fear for the foetus, compared to more than half of the participants in the intervention group. Such fear may lead to an increased number of CSs due to maternal requests. In [25], the authors stated that the causes of maternal requests for elective CSs were fear of labour pain, anxiety about foetal injury/death, fear of giving birth, urinary incontinence, and pelvic floor and/or vaginal injury. Our findings are consistent with a those of [26], which concluded that fear of complications and fear of death due to the operation were the most common causes of preoperative anxiety. In contrast, our findings are not compatible with those of [1], who reported the reason as fear of vaginal delivery following an adverse birth experience due to extended labour.

The mean STAI scores for the intervention and control groups in this study indicated no differences in the severity of anxiety between the groups before the educational sessions. However, after the educational sessions, there were notable changes in the severity of the anxiety. The overall means of the anxiety levels before the routine care and educational sessions in both the control and intervention groups were considerable. However, after the educational sessions, the overall mean anxiety level in the intervention group was lower than that before the sessions. Furthermore, the results showed that the mean differences in the STAI scores in terms of high overall anxiety between the intervention and control groups were significant, implying that the educational sessions effectively decreased anxiety levels among the pregnant women. Many studies have reported that pregnant women who attend appropriate educational sessions before their deliveries have lower anxiety levels compared to other pregnant women who receive no educational sessions [12,27,28]. In our study, we used the booklet not only for instructional purposes but also for reinforcement and future reference. This helped the pregnant women who attended the educational sessions experience less anxiety than those who were not given structured preoperative education and received only routine nursing care.

Additionally, the participants’ satisfaction levels after the preoperative educational sessions in the intervention group were high, with several women reporting being extremely satisfied. Previous studies have also shown the effectiveness of educational sessions before delivery in reducing the anxiety levels of pregnant women [7,29]. A similar study using STAI trait scores as a discriminator showed that individuals with median scores were prone to developing anxiety about CSs, which required interventions [2]. Regarding vital signs, there were significant differences in pulse, respiratory rate, temperature, and blood pressure before and after the educational sessions between the intervention and control groups. Only body temperature was not significantly different before or after the educational sessions. These findings are supported by studies conducted by [13] and [28]. In addition, Ref. [1] reported a reduction in anxiety levels and vital signs. Our findings are also consistent with those of [30], which revealed that surgical patients who received educational sessions had comparatively normal vital signs compared to those who received only routine care.

This study had some limitations; the inclusion criteria for this study limited the selection of cases, meaning that some cases in the hospital were excluded, which reduced the sample size and prolonged the data collection period. Hence, the study results cannot be generalised to a wider population.

## 5. Conclusions

This study highlighted the importance of conducting preoperative educational sessions for women undergoing CSs. Providing educational sessions together with a booklet containing information about CS surgery and its advantages and disadvantages, as well as instructions for aftercare following a CS, can help reduce preoperative anxiety, improve patient outcomes, and enhance patients’ involvement in their care and decision-making.

## Figures and Tables

**Table 1:** Participants’ sociodemographic characteristics.

Sociodemographic Characteristics	Intervention Group (n = 25)		Control Group (n = 25)		Test of Sig.	*p*
	No.	%	No.	%		
**Age (Years)**						
<30	15	60.0	15	60.0	χ^2^ = 0.968	^MC^ *p* = 0.663
30–35	8	32.0	6	24.0		
≥35	2	8.0	4	16.0		
Min.–Max.	21.0–35.0		23.0–43.0		*t* = 1.000	0.322
Mean ± SD	28.56 ± 4.13		29.80 ± 4.62			
**Education level**						
Elementary	0	0.0	0	0.0	χ^2^ = 2.122	0.145
Intermediate/Secondary	12	48.0	7	28.0		
University	13	52.0	18	72.0		
**Marital status**						
Married	24	96.0	22	88.0	χ^2^ = 1.087	^FE^ *p* = 0.609
Divorced	1	4.0	3	12.0		
**Occupation**						
Housewife	18	72.0	13	52.0	χ^2^ = 2.122	0.145
Working	7	28.0	12	48.0		

χ^2^ = chi-squared test, MC = Monte Carlo test, FE = Fisher’s exact test, *t* = Student’s *t*-test. *p* = *p* value for comparing the two groups.

**Table 2:** Obstetric and clinical data for the intervention and control groups.

Obstetric and Clinical Data	Intervention Group (n = 25)		Control Group (n = 25)		χ^2^	*p*
	No.	%	No.	%		
**Gravida**						
1–2	13	52.0	15	60.0	1.459	^MC^ *p* = 0.538
3–4	7	28.0	8	32.0		
5+	5	20.0	2	8.0		
**Parity**						
None	10	40.0	10	40.0	1.528	^MC^ *p* = 0.866
1–2	11	44.0	10	40.0		
3–4	3	12.0	5	20.0		
5+	1	4.0	0	0.0		
**Number of living children**						
None	10	40.0	10	40.0	0.100	^MC^ *p* = 1.000
1–2	11	44.0	11	44.0		
3–4	4	16.0	4	16.0		
**Previous methods of delivery**						
Vaginal birth	15	60.0	15	60.0	0.000	1.000
Caesarean birth	0	0.0	0	0.0		
None	10	40.0	10	40.0		
**Number of abortions**						
None	14	56.0	18	72.0	1.957	^MC^ *p* = 0.388
1–2	10	40.0	7	28.0		
3–4	1	4.0	0	0.0		

χ^2^ = chi-squared test, MC = Monte Carlo test, *p* = *p* value for comparing the two groups.

**Table 3:** Comparison of pre- and post-intervention means for overall anxiety levels and vital signs between the intervention and control groups.

Overall Anxiety and Vital Signs		Intervention Group (n = 25)	Control Group (n = 25)	*t*	*p*
**Overall Anxiety**					
Mean ± SD	**Pre**	65.68 ± 10.69	68.0 ± 9.88	0.797	0.429
2. Mean ± SD	**Post**	38.28 ± 9.31	69.36 ± 10.59	11.020 *	<0.001 *
***p*** **_0_**		<0.001 *	0.365		
**Temperature (c)**					
Mean ± SD	**Pre**	36.89 ± 0.24	36.94 ± 0.25	0.753	0.455
2. Mean ± SD	**Post**	35.64 ± 6.04	36.99 ± 0.12	1.113	0.277
***p*** **_0_**		0.308	0.327		
**Pulse (b/min.)**					
Mean ± SD	**Pre**	93.00 ± 4.45	94.64 ± 3.63	1.428	0.160
2. Mean ± SD	**Post**	90.88 ± 4.28	96.12 ± 3.94	4.506 *	<0.001 *
***p*** **_0_**		0.036 *	0.081		
**Respiratory (c/min.)**					
Mean ± SD	**Pre**	19.64 ± 1.70	19.40 ± 0.96	0.614	0.543
2. Mean ± SD	**Post**	18.00 ± 1.55	20.96 ± 1.10	7.775 *	<0.001 *
***p*** **_0_**		<0.001 *	<0.001 *		
**Blood pressure (mmHg)**					
**Systolic**					
Mean ± SD	**Pre**	137.72 ± 3.86	137.12 ± 3.26	0.594	0.555
2. Mean ± SD	**Post**	129.08 ± 3.20	136.28 ± 3.61	7.460 *	<0.001 *
***p*** **_0_**		<0.001 *	0.290		
**Diastolic**					
Mean ± SD	**Pre**	98.08 ± 2.83	96.36 ± 5.77	1.338	0.190
2. Mean ± SD	**Post**	87.12 ± 5.44	96.32 ± 4.45	6.543 *	<0.001 *
***p*** **_0_**		<0.001 *	0.976		

*t* = Student’s *t*-test, *p*_0_ = *p* value for the paired *t*-test for the pre- and post-intervention comparison, *p* = *p* value for comparing the two groups, * = statistically significant at *p* ≤ 0.05, before and after routine care in the control group, and before and after educational sessions in the intervention group.

**Table 4:** Mean STAI scores for the intervention and control groups.

STAI		Intervention Group (n = 25)	Control Group (n = 25)	*t*	*p*
Overall anxiety					
Mean ± SD	Before	65.68 ± 10.69	68.0 ± 9.88	0.797	0.429
Mean ± SD	After	38.28 ± 9.31	69.36 ± 10.59	11.020 *	<0.001 *
*p*_0_		<0.001 *	0.365		

*t* = Student’s *t*-test, *p*_0_ = *p* value for the paired *t*-test for pre- and post-intervention comparison, *p* = *p* value for comparing the two groups, * = statistically significant at *p* ≤ 0.05, before and after routine care in the control group, and before and after sessions in the intervention group.

**Table 5:** STAI levels of the intervention and control groups.

STAI	Intervention Group (n = 25)				Control Group (n = 25)				χ^2^ (*p*_1_)	χ^2^ (*p*_2_)
	Before		After		Before		After			
	No.	%	No.	%	No.	%	No.	%		
Overall anxiety										
Low	3	12.0	23	92.0	1	4.0	2	8.0	1.087 (^FE^ *p* = 0.609)	35.280 * (<0.001 *)
High	22	88.0	2	8.0	24	96.0	23	92.0		
***p*_0_**	<0.001 *				1.000					

χ^2^ = chi-squared test, *p*_0_ = *p* value for McNamara test for pre- and post-intervention comparison, *p*_1_ = *p* value for comparing the two groups pre-intervention, *p*_2_ = *p* value for comparing the two groups post-intervention, * = statistically significant at *p* ≤ 0.05.

**Table 6:** Women’s satisfaction with the preoperative educational sessions and overall anxiety in the intervention group.

Women’s Satisfaction	Overall Anxiety			
	High (n = 2)		Low (n = 23)	
	No.	%	No.	%
Were you satisfied with the education before surgery?				
Satisfied	2	100.0	15	65.2
2. Very satisfied	0	0.0	8	34.8
χ^2^ (^FE^ *p*)	**1.023 (1.000)**			
Were you satisfied with the preoperative information provided to you in the booklet?				
Satisfied	2	100.0	10	43.5
2. Very satisfied	0	0.0	13	56.5
χ^2^ (^FE^ *p*)	**2.355 (0.220)**			

χ^2^ = chi-squared test, FE = Fisher’s exact test, *p* = *p* value for the association between different categories.

**Table 7:** Correlations between mean overall anxiety levels and vital signs for the intervention and control groups (n = 50).

Overall Anxiety and Vital Signs		Intervention Group (n = 25)	Control Group (n = 25)	*t*	*p*
**Overall Anxiety**					
- Mean ± SD	**Pre**	65.68 ± 10.69	68.0 ± 9.88	0.797	0.429
- Mean ± SD	**Post**	38.28 ± 9.31	69.36 ± 10.59	11.020 *	<0.001 *
***p*** **_0_**	**-**	<0.001 *	0.365	-	-
**Temperature (c)**					
- Mean ± SD	**Pre**	36.89 ± 0.24	36.94 ± 0.25	0.753	0.455
- Mean ± SD	**Post**	35.64 ± 6.04	36.99 ± 0.12	1.113	0.277
***p*** **_0_**	**-**	0.308	0.327	-	-
**Pulse (b/min.)**					
- Mean ± SD	**Pre**	93.00 ± 4.45	94.64 ± 3.63	1.428	0.160
- Mean ± SD	**Post**	90.88 ± 4.28	96.12 ± 3.94	4.506 *	<0.001 *
***p*** **_0_**	**-**	0.036 *	0.081	-	-
**Respiratory (c/min.)**					
- Mean ± SD	**Pre**	19.64 ± 1.70	19.40 ± 0.96	0.614	0.543
- Mean ± SD	**Post**	18.00 ± 1.55	20.96 ± 1.10	7.775 *	<0.001 *
***p*** **_0_**		<0.001 *	<0.001 *		
**Blood pressure (mmHg)**					
**Systolic**					
- Mean ± SD	**Pre**	137.72 ± 3.86	137.12 ± 3.26	0.594	0.555
- Mean ± SD	**Post**	129.08 ± 3.20	136.28 ± 3.61	7.460 *	<0.001 *
***p*** **_0_**		<0.001 *	0.290		
**Diastolic**					
- Mean ± SD	**Pre**	98.08 ± 2.83	96.36 ± 5.77	1.338	0.190
- Mean ± SD	**Post**	87.12 ± 5.44	96.32 ± 4.45	6.543 *	<0.001 *
***p*** **_0_**		<0.001 *	0.976		

*t* = Student’s *t*-test, *p*_0_ = *p* value for the paired *t*-test for pre- and post-intervention comparison, *p* = *p* value for comparing the two groups, * = statistically significant at *p* ≤ 0.05, before and after routine care in the control group, and before and after educational sessions in the intervention group.

**Table 8:** Correlations among mean overall anxiety, post-educational session, sociodemographic characteristics, and obstetric data for the intervention and control groups.

Sociodemographic Characteristics and Obstetric Data	Overall Anxiety		*t*	*p*
	Intervention Group (n = 25)	Control Group (n = 25)		
	Mean ± SD	Mean ± SD		
**Age (Years)**				
<30	37.73 ± 8.73	66.13 ± 12.25	7.311 *	<0.001 *
30–35	39.25 ± 11.95	76.50 ± 1.87	7.492 *	<0.001 *
≥35	38.50 ± 0.71	70.75 ± 5.91	7.260 *	0.002 *
**Education level**				
Primary and Secondary	36.08 ± 2.78	74.57 ± 4.50	23.212 *	<0.001 *
University	40.31 ± 12.53	67.33 ± 11.65	6.177 *	<0.001 *
**Marital status**				
Married	42.04 ± 24.31	57.41 ± 31.23	1.871	0.068
Divorced	30.0	30.33 ± 0.58	0.500	0.667
**Occupation**				
Housewife	39.56 ± 10.61	73.00 ± 4.28	10.710 *	<0.001 *
Working	35.00 ± 3.16	65.42 ± 13.87	7.281 *	<0.001 *
**Gravity**				
1–2	35.69 ± 2.56	66.20 ± 12.35	9.340 *	<0.001 *
3–4	40.00 ± 12.71	74.63 ± 3.81	7.368 *	<0.001 *
5+	42.60 ± 14.40	72.00 ± 8.49	3.340 *	0.036 *
**Parity**				
None	35.60 ± 2.67	69.10 ± 7.23	13.736 *	<0.001 *
1–2	41.45 ± 13.39	68.10 ± 15.04	4.297 *	<0.001 *
3–4	37.33 ± 2.08	72.40 ± 5.59	12.634 *	<0.001 *
5+	33.0	–	–	–
**Number of abortions**				
None	35.36 ± 2.59	67.50 ± 11.78	11.229 *	<0.001 *
1–2	42.30 ± 13.78	74.14 ± 4.30	5.865 *	<0.001 *
3–4	39.0	–	–	–

*t* = Student’s *t*-test, *p* = *p* value for the associations between different categories, * = statistically significant at *p* ≤ 0.

## Data Availability

The data of this study are available from the corresponding author, upon reasonable request.
